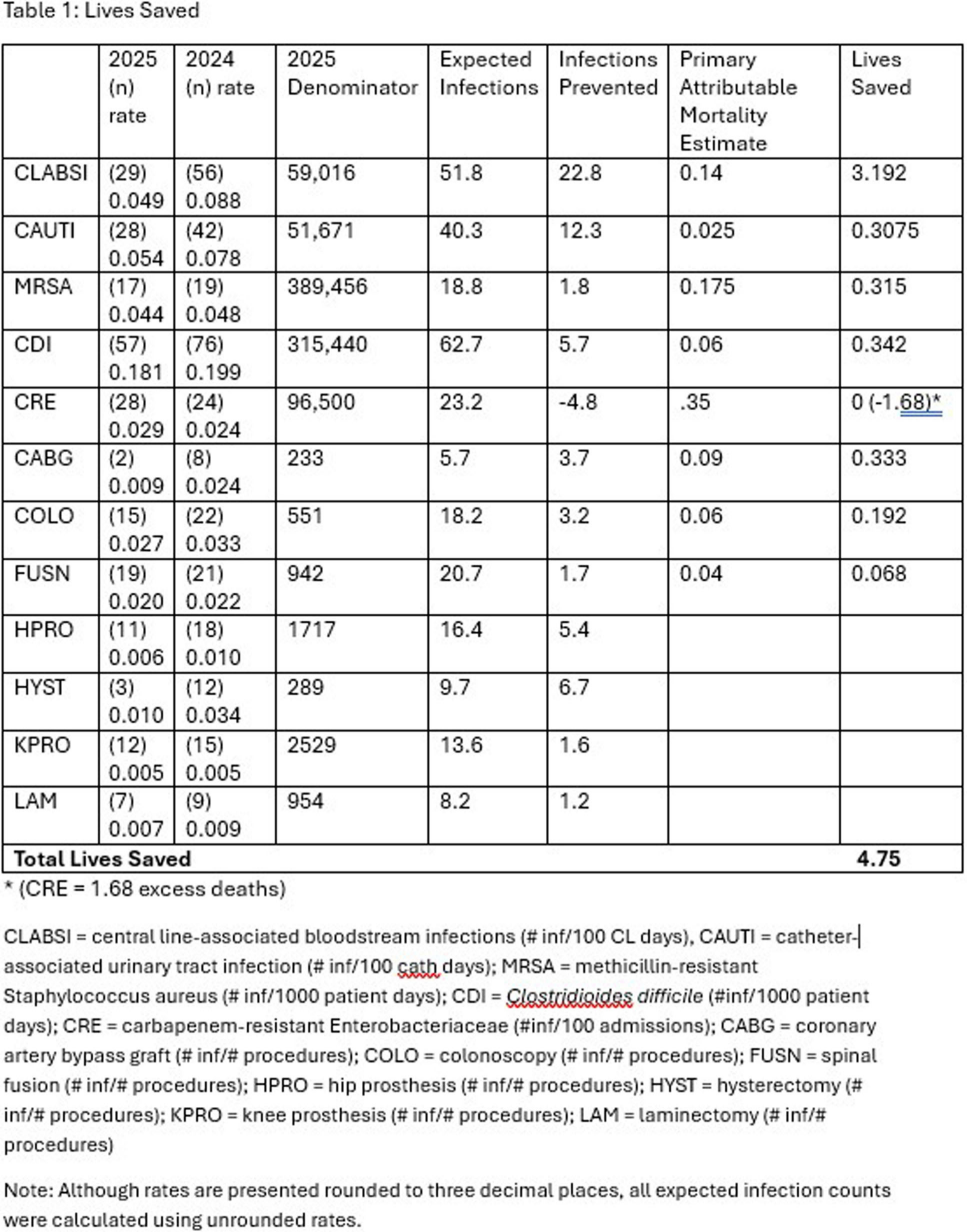# 324 What the Drains Reveal: Wastewater Surveillance of Multidrug-Resistant Organisms in Two Hospitals

**DOI:** 10.1017/ash.2026.10670

**Published:** 2026-06-23

**Authors:** Mackenzie Kross, Stephany Frey, Melissa Bronstein, Emil Lesho

**Affiliations:** 1 Rochester Regional Health

## Abstract

**Background:** In 2026, demonstrating the value of healthcare epidemiology and infection prevention is critical amid vaccine and science denialism and federal funding cuts. As hospitals face increasing financial pressures, executives may question sustained investment in healthcare epidemiologists and infection preventionists. Clinicians and nurses may also disengage from pay-for-performance or value-based initiatives. This project aimed to present a practical method for quantifying program outcomes for senior leadership, expressed as lives saved and costs avoided. **Methods:** Twelve types of healthcare-associated infections (HAI), including device-associated, laboratory, and surgical site infections—were identified across an eight-hospital health system in upstate New York using standardized CDC National Healthcare Safety Network definitions. Attributable mortality estimates were derived from published evidence. In the absence of improvement, infection rates were assumed stable year to year. Rates (R) from 2024 were multiplied by corresponding 2025 denominators (device days, patient days, or surgeries) (PD2025) to calculate expected infections (EI). Infections prevented (IP) were calculated as IP2025 = EI2025 – OI2025, where OI represents observed infections. To estimate total lives saved (TS) in 2025 compared with 2024, infections prevented (IP2025) were multiplied by the attributable mortality (AM) of each infection type to obtain lives saved (LS), then summed: IP20251 ՠAM1 = LS1; TS = ?LS. One-way sensitivity analyses across reported mortality ranges were performed using R. Cost avoidance was determined using facility-specific HAI costs and nationally reported cost ranges. **Results:** In 2025, 67 HAIs were prevented, corresponding to about five additional lives saved compared with the previous year (Table). The method also allowed estimation of costs and bed days saved. In our health system, each central line–associated bloodstream infection (CLABSI) and catheter-associated urinary tract infection (CAUTI) adds $97,588 and $61,939 in excess costs and 16.9 and 8.8 additional bed days, respectively. Using these and other adjusted HAI cost estimates for 2025, prevention of 67 infections yielded approximately $3.1 million in cost avoidance and 842 fewer inpatient bed days. **Conclusion:** Expressing outcomes as lives saved resonates more with executives and clinicians than abstract performance metrics. Because overuse of preventive measures (including unnecessary isolation) can cause collateral harm, prioritizing mortality outcomes is more relevant than pursuing reductions in rates or standardized infection ratios of all HAI types at any cost. This practical, generalizable method enables facilities, including those with limited research infrastructure, to quantify the impact of infection prevention and control and highlights the substantial effect of carbapenem-resistant Enterobacterales on overall mortality.

**Figure:**